# Usability Evaluation and Perceived Performance of the MoonWalking^®^ Insole in Safety Footwear

**DOI:** 10.3390/s26092668

**Published:** 2026-04-25

**Authors:** Pedro Castro-Martins, Arcelina Marques, Luís Pinto-Coelho, Mário Vaz

**Affiliations:** 1 CIETI, ISEP, Polytechnic of Porto, rua Dr. António Bernardino de Almeida, 4249-015 Porto, Portugal; 2 Faculty of Engineering, University of Porto, rua Dr. Roberto Frias, 4200-465 Porto, Portugal; 3 INEGI (Institute of Science and Innovation in Mechanical and Industrial Engineering), 4200-465 Porto, Portugal; 4 INESC-TEC, Centre for Robotics in Industry and Intelligent Systems, 4200-465 Porto, Portugal

**Keywords:** ergonomics, perceived comfort, plantar pressure, pneumatic insole, pressure offloading, safety footwear

## Abstract

**Highlights:**

**What are the main findings?**

- Pneumatic insole integration significantly improved perceived cushioning, foot fit, and overall comfort in safety footwear.
- Pressure-stabilization and offloading functions were consistently perceived across all plantar regions.

**What are the implications of the main findings?**

- Pneumatic adaptive pressure control is a viable engineering approach to enhance the ergonomic performance of safety footwear.
- The system demonstrates a perceived potential for mitigating plantar load in occupational standing and load-handling tasks.

**Abstract:**

Prolonged standing and repetitive lifting are routine occupational stressors that elevate plantar pressures across workers. In those with diabetes, these demands represent additional risk factors for diabetic foot pathology, highlighting the need for ergonomic interventions beyond standard safety footwear. This study evaluated the perceived ergonomic performance of the MoonWalking^®^ insole, a novel adaptive pneumatic system designed for real-time pressure stabilization and offloading when integrated into safety footwear. A comparative experimental protocol tested two conditions: safety footwear with the manufacturer’s original insole and the same footwear with the MoonWalking prototype. Twenty participants assessed perceived comfort using a VAS and binary ergonomic questionnaires. The results showed statistically significant improvements in perceived cushioning, foot fit, and overall comfort when using the MoonWalking insole. Participants consistently identified pressure-stabilizing and offloading functions across all plantar regions, indicating that adaptive pressure control was clearly perceptible. No pain or movement restrictions were reported. Although perceived fatigue did not reach statistical significance, a decreasing trend was observed. A slight reduction in intention to reuse the footwear occurred with the prototype, possibly due to its increased weight. These findings provide evidence that integrating an adaptive pneumatic insole into safety footwear may improve plantar pressure redistribution and user comfort.

## 1. Introduction

Safety footwear is an essential element of personal protective equipment across multiple industrial sectors, designed to ensure mechanical strength, stability, and protection against physical, chemical, and electrical hazards. However, compliance with regulatory requirements often implies compromises in terms of ergonomics, sole flexibility, and the ability to adapt to the morphology of the foot [1,2,3,4]. Hence, usability is an essential factor in the development and validation of user-oriented technological solutions, particularly in real-world contexts where comfort, functionality, and acceptance directly determine adoption and impact. In the field of functional footwear, and, in particular, safety footwear, the evaluation of usability is especially relevant, since this type of footwear is often used for long and continuous periods in demanding occupational settings, and is sometimes associated with discomfort, structural rigidity, limited cushioning capacity, and increased fatigue in the foot [5]. These factors can contribute to biomechanical overload of the foot, musculoskeletal pain, and reduced performance of the user throughout their working day [6,7,8,9] and are especially significant in workers who remain standing for long periods or are subject to repetitive loads [10,11]. This becomes even more critical in populations with changes in plantar sensitivity or with greater vulnerability to pressure injuries, as is the case with people with diabetes and those diagnosed with diabetic foot [12].

Diabetic foot condition is a frequent and clinically relevant complication of diabetes, characterized by neuropathic and vascular changes that compromise protective skin sensitivity, tissue integrity, and healing ability in the event of injury [13]. Associated with these factors, most plantar lesions that occur in people with diabetic foot are triggered by repeated plantar pressures that give rise to pressure ulcers [14]. It is estimated that between 19% and 34% of people with diabetes will develop plantar ulcers during their working life. This entails serious clinical consequences that include difficulty in healing, high risk of infection, and, in advanced cases, the use of minor or major amputation. These consequences result in a direct impact on individual mobility and absenteeism from work, which in turn entail a heavy burden on the economy and health systems [15,16,17,18,19]. When pressure thresholds and measurement technologies are well established [20,21], it is essential to develop adequate measures to mitigate risks.

In the workplace, workers with diabetes who wear safety footwear may be at increased risk of developing higher plantar pressure points, calluses, and skin lesions. Due to a loss of protective skin sensitivity, these people often do not perceive discomfort or mechanical aggression early [22,23]. Thus, strategies that promote better redistribution of plantar pressures, dynamic adaptation to the foot, and the reduction in localized pressure peaks are of particular interest not only from an ergonomic point of view, but also from the perspective of active prevention [24,25,26,27,28]. For this purpose, authors developed the MoonWalking^®^ insole (with previously published methodology and results [29]), which comprises a pair of insoles that employ a pneumatic system with dynamic control, representing an innovative technological approach with the potential to address the challenges described above. By redistributing plantar pressure through localized pressure control and continuous adaptation to the plantar region’s morphological characteristics, this device can reduce pressure points, improve cushioning, enhance foot fitting, and improve perceived stability during gait and orthostatism [29]. Although the present study focuses on healthy workers rather than a clinical diabetic population, analyzing the usability and functional perception of this type of solution in a real work context is an essential step to assess its future adequacy and potential acceptability among more vulnerable groups, such as workers with diabetes, where adherence to preventive footwear is critical [30,31].

The present study follows the authors’ recently published work [29], in which it addresses the development and validation of the MoonWalking prototype, a system for active and controlled relief of plantar pressure. In this context, the objective of the present study is to conduct an exploratory evaluation of the MoonWalking pneumatic insole’s usability when integrated into safety footwear, using, as a baseline, a similar shoe with a regular insole (as provided by the manufacturer).

The rest of this paper is organized as follows: Section 2 describes the materials and methods, including the study design, participant characteristics, description of the safety footwear and the MoonWalking system, the experimental protocol, the assessment instruments, and the statistical analysis. Section 3 presents the results obtained from the experimental evaluation. Section 4 discusses the main findings in the context of ergonomic performance and the system’s functionalities for plantar pressure redistribution, as well as addressing the study’s limitations. Finally, Section 5 provides the main conclusions of the study and outlines possible future research directions.

## 2. Materials and Methods

It was intended to collect preliminary indicators on perceptions of global comfort, cushioning, gait stability, adaptation to the foot, perceived temperature, and fatigue, as well as to evaluate the ergonomic acceptance and perception of the dynamic functionalities of the system, namely the stabilization and redistribution of plantar pressures. The choice of safety footwear as a test platform is justified by its high mechanical demand and its relevance in an occupational context, allowing the evaluation of the perceived performance of the MoonWalking insole in conditions close to real use. The methodology and materials used will be described in detail in the next subsections.

### 2.1. Study Design

An observational, experimental, and exploratory usability study with a crossover design was carried out. It included evaluating perceptions of comfort, functionality, and acceptance of the MoonWalking system, a dynamic pneumatic insole integrated into safety footwear. The exploratory nature of the study stems from the lack of prior data on the use of this pneumatic insole in a real-world context. This study aims to identify trends and early insights that will serve as a basis for future research in broader work contexts.

Two experimental conditions were used, and participants served as their own controls, reducing inter-individual variability and increasing the robustness of comparisons, particularly relevant in exploratory studies with small samples. The study included two conditions tested at different times and in random order of application: (i) use of safety footwear equipped with the manufacturer’s original insole (control condition) and (ii) use of the same footwear model equipped with the MoonWalking pneumatic insole (experimental condition). After each condition, the participants answered the same set of assessment instruments, allowing direct comparison between the two situations. The present study was approved by the Ethics Committee of the School of Health of the Polytechnic of Porto and was assigned registration no. CE0077F.

### 2.2. Participants

The sample consisted of adult volunteer participants recruited for convenience from the community and from workers who regularly use personal protective equipment in their professional activities. Inclusion and exclusion criteria were defined to ensure sample homogeneity and participant safety. The inclusion criteria included: (i) age equal to or greater than 18 years; (ii) regular use of safety footwear in a work context or sufficient level of literacy on the use of personal protective equipment; (iii) shoe size compatible with the tested insole (EU 43/44); and (iv) absence of active skin lesions on the feet. Participants were excluded if they met the following criteria: (i) structural deformities of the foot; (ii) report of some diagnosed foot, musculoskeletal, neurological, or vascular pathology; (iii) any clinical condition that could interfere with gait, plantar perception, or safety during the experimental protocol; and (iv) body mass greater than 120 kg. All participants provided informed consent before their participation, in accordance with the ethical principles of the Declaration of Helsinki and the General Data Protection Regulation (GDPR, Regulation (EU) 2016/679).

### 2.3. Safety Shoes and Moonwalking^®^ System

The safety footwear used in both experimental conditions is part of the X-COM line, model X-506, of the TOWORKFOR^®^ brand produced by AMF-Safety Shoes (Guimarães, Portugal). It is a work shoe with an S3 ESD SRC classification, featuring a non-slip sole, puncture resistance, impact protection, and electrostatic dissipation. This model was selected because it is representative of the safety footwear widely used in industrial and work settings, ensuring uniformity and comparability across the tested conditions. In the control condition, the footwear was used with the manufacturer-supplied insole. In the experimental condition, this insole was replaced by the pneumatic MoonWalking insole.

**Figure 1:**
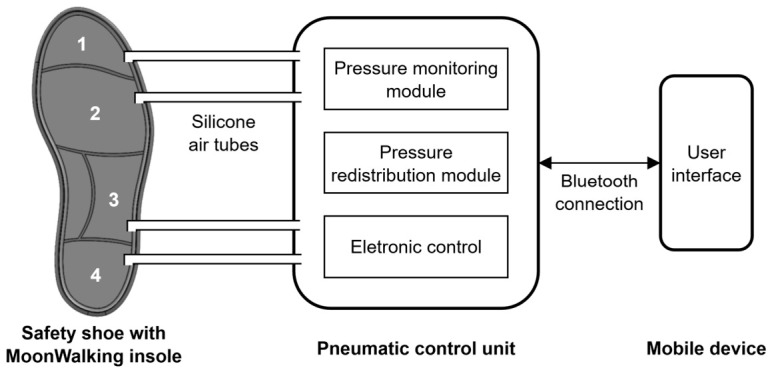
System architecture overview and MoonWalking insole with four action zones: (1) toes; (2) metatarsals; (3) lateral-midfoot; and (4) heel.

**Figure 2:**
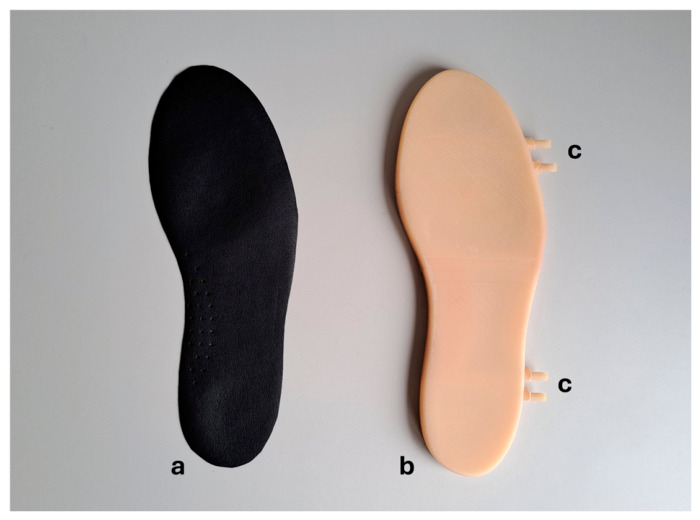
Components that make up the pneumatic insole size EU 43/44: (a) microfiber upper finish, (b) pneumatic rubber insole with air cells, and (c) four connection points for pneumatic tubes.

The MoonWalking prototype consists of an active pneumatic insole designed to monitor and redistribute plantar pressure inside the shoe (size EU 43/44) during walking, with a special focus on preventing injuries associated with the diabetic foot. The insole integrates air cells whose inflation levels can be adjusted in a controlled manner, allowing pressure to be regulated by zones and reducing pressure peaks at critical points of the foot, such as the toes, metatarsals, lateral-midfoot, and heel [29]. For better understanding, a high-level diagram of the MoonWalking system’s general architecture is presented in Figure 1. In Figure 2, the parts that make up the pneumatic insole coupled to the inside of the shoe are shown.

**Figure 3:**
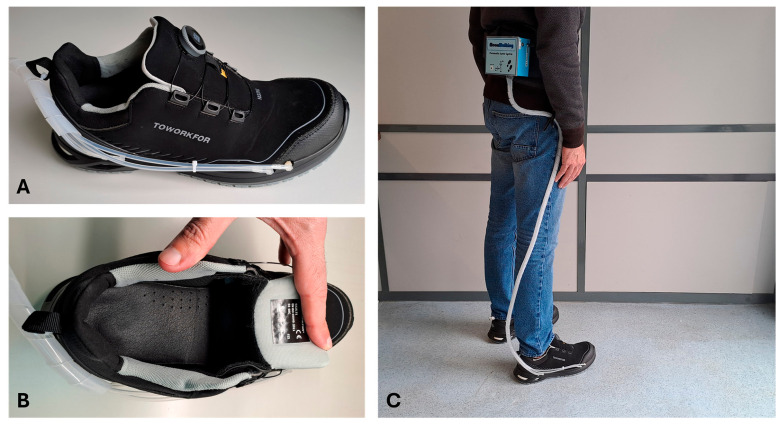
Safety shoe (TOWORKFOR, X-506) used in the usability study equipped with the pneumatic insole system (**A**), view of the inside of the shoe (**B**), and participant wearing it during one of the tests with the MoonWalking system connected (**C**).

In previous work [29], authors evaluated the MoonWalking system under static and dynamic conditions and demonstrated significant reductions in plantar pressure by redistributing pressure throughout the plantar region, thereby eliminating critical points causing high-pressure peaks. The pressure values were validated by comparison with a commercial reference system (pedar^®^), showing an agreement between 91% and 98%, depending on the region of the foot and the mode of operation [29]. Figure 3 illustrates the safety footwear used in this usability study and a participant using it during one of the tests with the MoonWalking system connected.

### 2.4. Experimental Protocol

Each participant performed two experimental sessions corresponding to the two testing conditions, in a random order to minimize learning effects and their consequent influence on the answers. In each session, the participant wore the corresponding pair of footwear (safety shoes with the original insole vs. safety shoes with the MoonWalking insole) for approximately 1 h, performing normal activities in their usual work context, including walking and standing.

The protocol was conducted in controlled conditions, aiming to replicate similar conditions across all tests performed by the various participants, ensuring at least one gait and one standing period. Between each experimental session, a 15 min rest interval was provided, and at the end of each session, participants completed the usability and subjective perception assessment instruments for the tested condition. The 15 min interval between the two test conditions was strategically implemented, as it allowed participants to achieve sensory stabilization, maintaining sufficient immediate tactile memory for an accurate comparative assessment between the prototype and the control shoe. To further mitigate potential residual effects, order biases, or learning effects, the sequence of conditions was randomized for each participant, ensuring that any residual influence was distributed equally among them. Additionally, after using the MoonWalking insole, a specific test was conducted to assess the perception of the system’s dynamic functionality, followed by the corresponding functional questionnaire.

The functionalities of the MoonWalking system include the stabilization and offloading of localized pressure and consequent redistribution of plantar pressure across the four active zones (toes, metatarsals, lateral-midfoot, and heel). Stabilization consists of a pressure adjustment in all air cells of the pneumatic insole so that its structure accommodates the foot morphology, immediately after putting on the shoe. Offloading and redistribution consist of the localized pressure offload (in one of the four zones) when the pre-configured pressure threshold is reached, and, consequently, the plantar pressure will be redistributed throughout the new contact area, thus eliminating the pressure point originated, or minimizing the magnitude of the pressure at that location. In this second feature, when the system is operating in active/automatic mode, it can perform this procedure as many times as necessary until it reaches the defined threshold or reaches the minimum operating air volume. To assess the perception of the system’s functionalities, these actions were simulated, forcing the system to act as if a pressure event had occurred above a certain defined threshold. During the pressure stabilization process, participants were kept standing in a static mode. During the pressure-offloading process and its redistribution, participants were standing or walking.

To ensure that all participants were exposed to the same mechanical stimuli within the experimental timeframe, the activation of the system’s specific functions was induced in a controlled and safe manner. This standardized exposure was necessary because, in a natural walking or standing posture with healthy participants, the pressure thresholds required for automatic activation might not be reached consistently. To safeguard the integrity of the subjective data, a single-blind approach was adopted: participants were not informed when an activation was about to occur. After each potential event, subjects were questioned about their perception of any pneumatic action. Furthermore, to control response bias and ensure the veracity of the reports, control questions were asked during periods where the system was intentionally inactive. This allowed us to verify the participants’ ability to accurately distinguish between pneumatic interventions of the system and other normal variations in footwear use.

Due to limitations on the number of prototypes, this study evaluated only the right foot; however, to provide stability for the participant, an equal pneumatic insole was used in the left footwear. Although the left insole was not connected to the pneumatic control system and only acted passively, it was pressurized correctly and sealed with the pre-established support pressure for each cell (pre-configured by the system), and the same for both feet at the beginning of the test.

### 2.5. Assessment Instruments

Participants’ subjective perceptions were assessed using validated instruments commonly employed in usability and ergonomic footwear studies. The primary tool was an adapted Visual Analogue Scale (VAS) [32], providing the participant with a range of 0 to 10 points to assign their classification, with 0 corresponding to the minimum perception and 10 to the maximum perception of the evaluated parameter. The VAS facilitates direct statistical comparisons between conditions and the detection of subtle improvements in adaptive systems such as the MoonWalking prototype. Participants assigned ratings for the following parameters: overall comfort level, cushioning, stability during gait, adaptation of the insole to the foot’s morphology, perceived temperature, and fatigue at the end of the use period. This instrument allowed a direct quantification of subjective perception in both experimental conditions. In addition, binary questions (“Yes” or “No” answers) were included to assess ergonomic and acceptance outcomes, namely the presence of movement limitations, the occurrence of localized discomfort or pain, and the intention to use the footwear with the respective insole in the future.

After using the pneumatic insole, a functionality questionnaire was also administered, specifically developed to assess perceptions of the mechanical actions of the MoonWalking system. This questionnaire focused on perceptions of stabilization and pressure offload across different plantar regions (toes, metatarsals, lateral-midfoot, and heel), and responses were recorded as binary (“Yes” or “No”). Finally, participants were invited to provide spontaneous observations about their user experience, which were collected and subsequently analyzed qualitatively, allowing the identification of recurring themes and relevant aspects not captured by the subjective assessment instruments.

Although the present study focuses on subjective evaluation, the mechanical effectiveness of the prototype in redistributing loads was previously validated using shoe pressure mapping systems, demonstrating significant reductions in pressure peaks in critical areas [29]. The present usability analysis therefore aims to complement these technical data with the perspective of user perception.

### 2.6. Statistical Analysis

For the usability study, data analysis used descriptive and inferential statistics. The continuous variables obtained from the VAS are presented as means and standard deviations. Given the small sample size and the ordinal nature of the scales, the comparison between the two experimental conditions was conducted using the nonparametric Wilcoxon test for paired samples. The level of statistical significance was set at *p* < 0.01 to increase the precision of the results and the robustness of the conclusions, ensuring that the observed associations have high statistical confidence.

The answers to the binary questions and the functionality questionnaire were analyzed descriptively, with results presented as percentages of affirmative responses. The spontaneous qualitative observations of participants who wished to give their opinion were organized and categorized through thematic analysis, with the aim of identifying common patterns of perception among the participants. This thematic analysis process involved the complete transcription of the responses obtained, followed by open coding to identify and label significant segments of the comments. These initial codes were iteratively reviewed and grouped into transversal themes based on their similarity. The coding process was conducted by a single researcher and subsequently reviewed by the research team to ensure consistency and coherence in the interpretation.

## 3. Results

The sample consisted of 20 adult participants aged 18 to 65 years. Participants were recruited for convenience from the general community and from workers who regularly use safety footwear in the performance of their work duties, or who have adequate knowledge of personal protective equipment use. The previously defined inclusion and exclusion criteria were applied to ensure participant eligibility. All subjects provided informed consent before participation in the study. Table 1 presents the demographic and anthropometric characterization of the sample.

It is important to note that the sample consisted mainly of male participants (n = 18), with only 2 female participants included. This disparate distribution is not the result of an intentional sex-based selection criterion, but instead of the footwear size (EU 43/44) restriction imposed by the experimental protocol, which is often smaller in women, thereby conditioning eligibility for participation in the study.

Considering the analyzed dimensions indicated previously, Table 2 presents the mean values and respective standard deviations for each parameter across the two experimental conditions: safety footwear with the original insole (condition A) and with the MoonWalking insole (condition B). The mean difference between conditions (B − A) and the statistical significance value resulting from the paired comparison are also presented.

**Figure 4:**
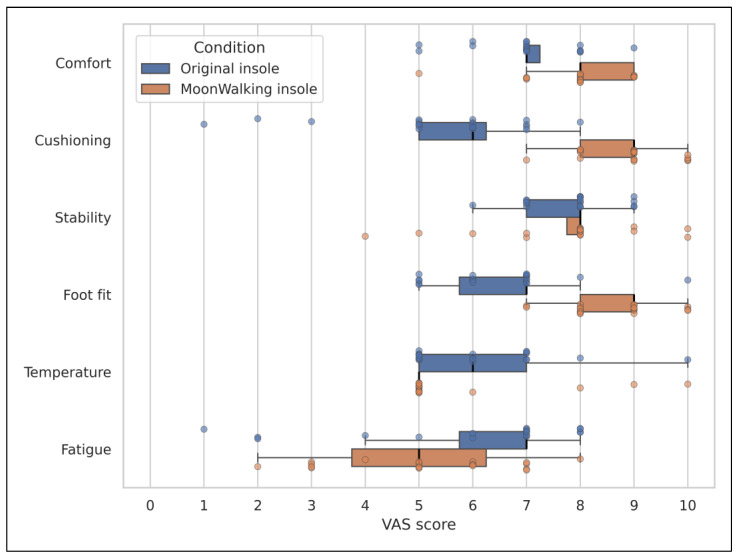
Distribution of VAS scores for perceived ergonomic parameters comparing the original insole and the MoonWalking insole. Boxplots represent the median and interquartile range, while individual points correspond to participant responses.

Among the parameters evaluated, statistically significant differences were observed in cushioning perception (*p* = 0.0001), foot fit (*p* = 0.0002), and comfort (*p* = 0.0073), with higher mean values in the MoonWalking insole condition. In all three cases, the mean VAS score reached 8 or higher, representing a considerable increase compared to the original insole condition, especially in cushioning and foot fit. The remaining parameters, such as stability during gait and perceived temperature, did not show statistically significant differences between the two conditions (*p* > 0.01), showing broadly similar perceptions. Regarding fatigue at the end of the use period, there was a mean reduction of 0.9 points on the VAS in the pneumatic insole condition; however, this difference did not reach significance (*p* = 0.1054). Figure 4 complements the information presented in Table 2 and shows the distribution of VAS scores for the perceived ergonomic parameters between the original insole and the MoonWalking insole.

To complement the quantitative evaluation obtained from the VAS, binary questions (“Yes” or “No”) were included to characterize the system’s ergonomic perception and acceptability. These questions allowed the identification of potential functional limitations not captured by the VAS, namely perceptions of movement restrictions, the occurrence of localized pain or discomfort, and the intention to use the shoe with the original insole and with the MoonWalking insole in the future. Table 3 presents the percentage distribution of affirmative responses for each experimental condition, allowing a direct comparison of the acceptance and tolerance of the MoonWalking insole with those of the manufacturer’s original insole.

Regarding ergonomic perception, no limitations in movement or localized pain were reported across any experimental conditions, suggesting good tolerance for both the original footwear and the MoonWalking system. Regarding the intention to use it again, 85% of participants indicated they would use the MoonWalking insole again, compared with 90% in the original insole condition. Although slightly lower than the control condition, this value shows a positive preliminary acceptance of the MoonWalking system in its current configuration. It is important to note that, given the small sample size, a single negative answer can significantly affect the results presented. However, in absolute terms, in the total sample (n = 20), three participants refused to wear the shoe with the MoonWalking insole again, and two would not wear it with the original insole. Therefore, only one participant stated that he would wear the shoe with the original insole again, but would not do so when equipped with the pneumatic insole. This decision was based on the concern that the shoe’s weight could be uncomfortable during a full workday.

After using the MoonWalking insole, participants also completed a specific functionality questionnaire to assess their perception of the pneumatic system’s mechanical actions. Two main dimensions were analyzed: (i) stabilization, understood as an adjustment of the pressure in all air cells of the pneumatic insole so that its structure accommodates the foot morphology, and (ii) localized pressure offloading, associated with the relief of pressure in a particular area and consequent redistribution of the load to the different regions of the foot. Table 4 presents the percentage of affirmative answers (“Yes”) regarding the perception of these functionalities in each plantar zone analyzed.

The functional evaluation of the MoonWalking system showed consistent perception of the stabilization and pressure offloading functionalities across all plantar regions analyzed, with 100% affirmative responses in both dimensions. These results, corroborated by the pressure values before and after activating the system’s functionalities, suggest that the mechanical actions induced by the pneumatic system were clearly perceptible to users, indicating a correspondence between the system’s technical behavior and the subjective user experience.

At the end of the experimental protocol, participants were asked to provide spontaneous observations about the user experience. The answers were analyzed qualitatively and grouped into thematic categories, allowing the identification of recurrent trends. Thematic analysis of spontaneous qualitative observations identified three central themes: (i) ergonomic performance, where comfort and cushioning were widely praised; (ii) user experience, highlighting the clear perception of pressure changes (stabilization and offloading) without any indication of interference or discomfort during or after actuation; and (iii) pneumatic insole design, where the extra weight of the prototype was identified as the main point for improvement for future iterations, with this being one of the main negative comments from participants who consider not using the prototype again. Regarding the safety footwear used, several participants reported that it tends to be less comfortable than conventional everyday footwear, mainly due to the greater weight and the slight increase in temperature during use, characteristics inherent to its function as safety footwear. In the MoonWalking insole condition, these perceptions were maintained because the shoe model remained the same. However, occasional comparisons between the two conditions were recorded, in which some participants reported a slight increase in the total weight of the shoe (beyond the weight of the original shoe), attributed to the integration of the pneumatic insole. In the participants who indicated that they would not use the prototype again, the increase in weight of the shoe with the integration of the pneumatic insole was identified as one of the conditions for not using the shoe with the system again. The slight weight increase of the prototype compared to the standard shoe was one of the features that participants expressed most in their free comments at the end of the evaluations. These comments were interpreted as constructive contributions for future optimizations of the MoonWalking system.

## 4. Discussion

The results show that, although the MoonWalking system is still in a prototype phase, participants reported globally acceptable levels across the evaluated parameters, with no reports of localized pain or movement restrictions. This is particularly relevant in the context of safety footwear, where structural and material requirements often tend to compromise comfort for the sake of protection, as suggested by Arceri et al. [33]. The fact that the pneumatic insole has maintained (in specific parameters, there were even improvements) the subjective perception in relation to the original insole, suggests that the system has an ergonomics compatible with a possible application in a work context, and may be an asset for workers subject to long periods in orthostatism or load handling. Although the protocol had a limited duration, obtaining positive usability and comfort indicators at this stage of proof-of-concept is an essential prerequisite to ensure user safety and acceptance in future longer exposure trials.

The statistically significant improvement in perception of cushioning and foot fit, as evidenced by VAS scores, reinforces the role of dynamic pressure adjustment in uniformity of plantar pressure distribution. The system’s ability to adapt the insole’s structure to the foot’s morphology appears to yield a clear perceptual experience for the user. This aspect was corroborated in the subsequent functional assessment, in which all participants identified stabilization and pressure-offloading actions across all plantar regions analyzed. The conscious perception of these functionalities is a relevant indicator of functional validity, as it evidences a correspondence between the system’s mechanical behavior and the user’s subjective experience. The perception of overall comfort also showed a statistically significant improvement, reinforcing the consistency of the positive effects observed in cushioning and foot fit. This result suggests that pneumatic adaptive control does not act only on isolated parameters but contributes in an integrated way to the overall user experience during walking. Improved comfort may be associated with a more homogeneous redistribution of plantar pressure and dynamic adaptation throughout the gait cycle, thereby reducing localized overload. To support these results, in the literature on VASs, a variation greater than 10% (1 point on a scale of 0 to 10) is frequently cited as the minimum clinically important difference [34]. In our study, among the parameters that proved to be statistically relevant, although comfort showed a positive variation of 10%, we observed improvements in parameters such as foot fit and cushioning that exceeded 2 VAS points (21% to 35% of the VAS, respectively), suggesting that the statistical significance achieved translates into a perceptible practical benefit for the user. In addition, the participants’ body mass range (55–90 kg) is a relevant factor in plantar pressure distribution; however, the positive perception of comfort, cushioning, and foot fit was consistent across this range, suggesting a preliminary versatility of the pneumatic system. Although the crossover design controlled this variable during the comparison, it is important to note that in future studies with larger groups, the correlation between body mass index (BMI) and the effect of the intervention on the effectiveness of plantar pressure redistribution and offloading should be investigated.

Although the perception of fatigue did not reach statistical significance, the observed decrease in mean trend remains consistent with the significant increase in overall comfort. It is plausible that, in longer-duration protocols or in a real occupational context, the combined effects of improvements in cushioning, foot fit, and comfort may translate into a greater reduction in plantar fatigue than with conventional safety footwear. The lack of statistical significance in fatigue reduction may be attributed to the limited exposure time (1 h), which may not have been sufficient to reach the fatigue threshold necessary for a clear differentiation. These data justify conducting longitudinal studies to evaluate possible cumulative effects associated with continued use of the pneumatic insole.

Regarding the intention to wear the shoes again, there was a slight decrease in the percentage of participants who stated they would wear the shoes with the MoonWalking insole again (85%) compared with the control condition (90%). This difference should be interpreted with caution. Firstly, the units tested also corresponded to academic validation prototypes. Secondly, in a small sample, a single negative answer has a relevant percentage impact on the results presented. In addition, it is important to consider the weight gain associated with integrating the pneumatic system. For size EU 44, the approximate mass of a conventional everyday shoe is around 450 g, while the safety footwear used in the study weighs 750 g; when combined with the MoonWalking insole, the total mass is 930 g. This increase may have influenced the overall perception of intention to wear this shoe again. For participants who indicated that they would not use the prototype again, weight gain was the main characteristic pointed out for this decision. The increase in mass (from 750 g to 930 g) represented an increase of approximately 24% in the weight of the footwear. Although this value influenced the perception of some users, the results suggest that improvements in cushioning and foot fit (around +3.5 and +2.1 points on the VAS scale, respectively) compensated, for most participants, for the additional effort associated with the weight of the prototype. However, in a future industrialized version integrating the MoonWalking insole during manufacturing, removing material from the original sole to accommodate the pneumatic insole will predictably keep the final weight close to the original safety shoe. Thus, aspects related to weight, aesthetics, and finish can be optimized without compromising the intended functionality, thereby enhancing the MoonWalking system’s acceptability.

### Study Limitations

Despite the promising results, the study has limitations that should be considered when interpreting the data. The evaluation was conducted exclusively on the right foot due to constraints in prototype production, which prevented a complete bilateral analysis. Regarding the sample, it included fewer female participants due to the available sizes of the prototype, limiting its representativeness. The small sample size implies less statistical power and may have greater sensitivity to individual variation, because a single negative response can have a significant impact, especially in binary analyses. However, the use of a crossover design allowed each participant to act as their own control, minimizing the influence of interindividual variability and providing greater robustness to the observed trends, despite the exploratory nature of the study. Finally, the short duration of the experimental protocol and the lack of control over the time of day when participants used the system do not allow the evaluation of possible cumulative effects associated with daily use in a real occupational context.

## 5. Conclusions

The integration of the MoonWalking system into safety footwear is particularly relevant in occupational safety and health, especially for workers subjected to long periods of standing or repetitive handling of loads. These conditions induce high plantar pressure, a known risk factor for discomfort and foot injuries, as well as long-term musculoskeletal problems. The results obtained establish a usability baseline necessary for future research in more demanding clinical or occupational contexts, suggesting that this technology may have potential for testing in vulnerable populations predisposed to pressure-related foot complications.

The statistical analysis of this study showed significant improvements in cushioning perception, foot fit, and overall comfort, reinforcing the consistency of the positive effects of adaptive pressure control. These results suggest that the dynamic redistribution of plantar pressure and the structural adaptation of the insole to the foot’s morphology contribute, in an integrated way, to improvements in the subjective experience during shoe use. The unanimous identification of the stabilization and pressure offloading functionalities further confirms that the system’s mechanical behavior is clearly perceptible to the user. In addition, no limitations in movement or localized pain were reported, and, in general, some indicators indicate good acceptability of the system in its current configuration.

Although the perception of fatigue did not reach statistical significance, the observed improvement is consistent with the classification of global comfort. The slight reduction in the intention to wear the shoe with the pneumatic system again should be interpreted in terms of the prototype nature and the increase in the weight of the shoe associated with the integration of the insole, but also in relation to the weight characteristic of a safety shoe of this nature. Overall, the results provide preliminary evidence that integrating an adaptive pneumatic system into safety footwear is technically feasible and may represent a promising strategy to minimize exposure to high plantar pressure in occupational contexts.

In conclusion, the results suggest that the MoonWalking system presents promising usability and that its adaptive pneumatic control can positively influence the user’s subjective experience. However, these findings should be interpreted considering the limitations of the sample studied, and their generalization to other genders, shoe sizes, and populations with pre-existing pathologies requires further studies using an optimized prototype and more diverse groups. Furthermore, while the usability results are encouraging, the translation of these benefits into objective indicators of biomechanical performance still needs to be established through further validation in prolonged use contexts.

### Future Direction

Future developments should focus primarily on expanding the range of available insole sizes beyond EU 43/44, allowing for a more representative sample and a more balanced distribution by gender. Increasing the sample size and stratifying participants by body mass index (BMI) and occupation/profession are essential steps to strengthen statistical power and reduce the impact of individual variations on the results. Secondly, the bilateral evaluation of the system is also a priority step to enable a more complete analysis of the user’s perception, allowing for the research of synchronous bilateral adaptation and its impact on gait symmetry and long-term postural stability. The duration of system usage time cannot be ignored in future studies, since a longer usage time allows the evaluation of dynamic changes in comfort and fatigue throughout the workday (for example, 8 h), as well as verifying the system’s durability.

In addition, the structural optimization of the system, namely, weight reduction through the industrial integration of the insole during the footwear manufacture, should further improve the acceptability and potential commercial application of the technology in the safety footwear market. After these steps, it will be essential to conduct longitudinal studies across a broader range of occupational contexts to evaluate the cumulative effects of prolonged daily use, specifically on comfort and plantar fatigue. Although the system has been tested on healthy individuals, the demonstrated offloading technology could also, in the future, be explored in at-risk populations, such as people with diabetic foot.

## Figures and Tables

**Table 1:** Demographic and anthropometric data of study participants.

Sample (n)	Sex		Age Range (Years)	Weight Range (kg)	Height Range (cm)	Foot/Shoe Size (EU Size)
	M	F				
20	18	2	18–65	55–90	165–185	43/44

**Table 2:** Mean VAS results (0 to 10) for the two experimental conditions. The comparison between conditions was performed using the Wilcoxon test for paired samples. Statistical significance was considered *p* < 0.01 (*).

Parameter (Perceived)	Original Insole (A) (VAS ± SD)	MoonWalking Insole (B) (VAS ± SD)	Mean Difference (B − A)	*p*-Value	Observed Trend
Comfort	7.0 ± 1.0	8.0 ± 1.0	+1.0	0.0073 *	Significant increase in perceived comfort
Cushioning	5.4 ± 1.7	8.9 ± 0.8	+3.5	0.0001 *	Significant increase in perceived cushioning
Stability	7.9 ± 0.8	7.8 ± 1.4	−0.1	0.9142	Similar perceived stability
Foot fit	6.5 ± 1.2	8.6 ± 0.9	+2.1	0.0002 *	Significant increase in perceived foot fit
Temperature	6.1 ± 1.3	5.7 ± 1.5	−0.4	0.0848	Similar perceived temperature
Fatigue	6.1 ± 2.1	5.2 ± 1.7	−0.9	0.1054	Decreased perceived fatigue, not significant

**Table 3:** Percentage of affirmative answers (“Yes”) in questions related to ergonomic perception and acceptability in the two experimental conditions.

Questions About Ergonomics	Original Insole “Yes” (%)	MoonWalking Insole “Yes” (%)
Have you noticed any limitations of movement?	0.0	0.0
Have you noticed any localized pain?	0.0	0.0
Would you wear these shoes again with this insole?	90.0	85.0

**Table 4:** Percentage of affirmative answers (“Yes”) regarding the perception of the stabilization and pressure offloading functionalities of the MoonWalking insole, during the activation of the pneumatic system. Affirmative responses corroborated by the pressure values before and after the action.

Plantar Zone	Perceived Pressure Stabilization			Perceived Pressure Offloading		
		Pressure (kPa) (Mean ± SD)			Pressure (kPa) (Mean ± SD)	
	“Yes” (%)	Before	After ^(a)^	“Yes” (%)	Before	After ^(b)^
Toes	100	10.8 ± 2.8	24.3 ± 6.7	100	10.6 ± 4.1	5.1 ± 1.6
Metatarsals	100	19.8 ± 6.8	23.2 ± 6.4	100	26.2 ± 9.8	18.4 ± 6.8
Lateral-midfoot	100	26.2 ± 8.5	24.5 ± 6.6	100	22.0 ± 5.8	16.3 ± 4.3
Heel	100	50.7 ± 8.4	24.0 ± 7.2	100	56.3 ± 9.9	42.3 ± 6.3

^(a)^ The pressure was distributed throughout the entire plantar region after the stabilization action; ^(b)^ The pressure decreased after the localized offloading action by zone.

## Data Availability

The original data and results obtained in this study are included in this article. If necessary, additional information can be requested from the corresponding author.

## Abbreviations

The following abbreviations are used in this manuscript:

BMI	Body Mass Index
ESD	Electrostatic Discharge (safety footwear classification)
EU	European Union
EU 44	European shoe size (in this case, size 44)
GDPR	General Data Protection Regulation
S3	Category 3; Level of protection in safety footwear (EN ISO 20345)
SRC	Slip Resistance Certification (safety footwear classification)
VAS	Visual Analog Scale

## References

[1] Krings B.M., Miller B.L., Chander H., Waldman H.S., Knight A.C., McAllister M.J., Fountain B.J., Smith J.W. (2018). Impact of occupational footwear during simulated workloads on energy expenditure. Footwear Sci..

[2] Chiou S.S., Turner N., Zwiener J., Weaver D.L., Haskell W.E. (2012). Effect of boot weight and sole flexibility on gait and physiological responses of firefighters in stepping over obstacles. Hum. Factors.

[3] Benjamin D., Ahram T., De Ru E., Choukou M.A., Abdi E., Gardan N., Boyer F.C., Regnault P., Taiar R. (2017). Comparison of FAP scores with the use of safety footwear and regular walking shoes. Theor. Issues Ergon. Sci..

[4] Ochsmann E., Noll U., Ellegast R., Hermanns I., Kraus T. (2016). Influence of different safety shoes on gait and plantar pressure: A standardized examination of workers in the automotive industry. J. Occup. Health.

[5] Alferdaws F.F., Ramadan M.Z. (2020). Effects of lifting method, safety shoe type, and lifting frequency on maximum acceptable weight of lift, physiological responses, and safety shoes discomfort rating. Int. J. Environ. Res. Public Health.

[6] Orr R., Maupin D., Palmer R., Canetti E.F.D., Simas V., Schram B. (2022). The Impact of Footwear on Occupational Task Performance and Musculoskeletal Injury Risk: A Scoping Review to Inform Tactical Footwear. Int. J. Environ. Res. Public Health.

[7] Copper A.W., Scharfbillig R., Nguyen T.P., Collins C. (2021). Identifying lower limb problems and the types of safety footwear worn in the Australian wine industry: A cross-sectional survey. J. Foot Ankle Res..

[8] Marr S.J., Quine S. (1993). Shoe concerns and foot problems of wearers of safety footwear. Occup. Med..

[9] Dobson J.A., Riddiford-Harland D.L., Bell A.F., Steele J.R. (2017). Work boot design affects the way workers walk: A systematic review of the literature. Appl. Ergon..

[10] Chander H., Garner J.C., Wade C., Knight A.C. (2017). Postural control in workplace safety: Role of occupational footwear and workload. Safety.

[11] Arceri A., Mazzotti A., Liosi S.G., Zielli S.O., Artioli E., Langone L., Traina F., Brognara L., Faldini C. (2024). Safety Footwear Impact on Workers’ Gait and Foot Problems: A Comparative Study. Clin. Pract..

[12] Mondal S., Lodh M., Sahoo S., Paul K., Biswas D., Krishna C., Parida A., Ganguly A., DasGupta R. (2025). Prevalence and predictors of infected diabetic foot ulcers (DFU) and DFU-related osteomyelitis amongst industrial workers wearing occupational safety footwear. Sci. Rep..

[13] Schaper N.C., van Netten J.J., Apelqvist J., Bus S.A., Fitridge R., Game F., Monteiro-Soares M., Senneville E., on behalf of the IWGDF Editorial Board (2024). Practical guidelines on the prevention and management of diabetes-related foot disease (IWGDF 2023 update). Diabetes Metab. Res. Rev..

[14] Bus S.A., Armstrong D.G., Gooday C., Jarl G., Caravaggi C., Viswanathan V., Lazzarini P.A., on behalf of the International Working Group on the Diabetic Foot (IWGDF) (2020). Guidelines on offloading foot ulcers in persons with diabetes (IWGDF 2019 update). Diabetes Metab. Res. Rev..

[15] Chou Y., Hou C., Wu C., Huang D., Tsai S., Liu T., Ding L., Chang C., Ou K., Chiu Y. (2022). Risk factors that predict major amputations and amputation time intervals for hospitalised diabetic patients with foot complications. Int. Wound J..

[16] Hicks C.W., Canner J.K., Mathioudakis N., Lippincott C., Sherman R.L., Abularrage C.J. (2020). Incidence and Risk Factors Associated with Ulcer Recurrence Among Patients with Diabetic Foot Ulcers Treated in a Multidisciplinary Setting. J. Surg. Res..

[17] Armstrong D.G., Boulton A.J.M., Bus S.A. (2017). Diabetic Foot Ulcers and Their Recurrence. N. Engl. J. Med..

[18] McDermott K., Fang M., Boulton A.J.M., Selvin E., Hicks C.W. (2023). Etiology, Epidemiology, and Disparities in the Burden of Diabetic Foot Ulcers. Diabetes Care.

[19] Edmonds M., Manu C., Vas P. (2021). The current burden of diabetic foot disease. J. Clin. Orthop. Trauma.

[20] Castro-Martins P., Marques A., Coelho L., Vaz M., Costa J.T. (2024). Plantar pressure thresholds as a strategy to prevent diabetic foot ulcers: A systematic review. Heliyon.

[21] Castro-Martins P., Marques A., Coelho L., Vaz M., Baptista J.S. (2024). In-shoe plantar pressure measurement technologies for the diabetic foot: A systematic review. Heliyon.

[22] Cavanagh P.R., Ulbrecht J.S., Apelqvist J., Stenstrom A., Kalpen A., Bus S. (2022). In-shoe plantar pressure threshold for the prevention of plantar ulcer recurrence. Diabetes.

[23] Stess R.M., Jensen S.R., Mirmiran R. (1997). The role of dynamic plantar pressures in diabetic foot ulcers. Diabetes Care.

[24] Jarl G., Rusaw D.F., Terrill A.J., Barnett C.T., Woodruff M.A., Lazzarini P.A. (2023). Personalized Offloading Treatments for Healing Plantar Diabetic Foot Ulcers. J. Diabetes Sci. Technol..

[25] Ntella S.L., Jeanmonod K., Civet Y., Koechli C., Perriard Y. (2022). Pressure Offloading Device for Diabetic Footwear Based on Magnetorheological Fluids. Proceedings of the 2022 25th International Conference on Electrical Machines and Systems (ICEMS), Chiang Mai, Thailand, 29 November–2 December 2022.

[26] Snyder R.J., Lanier K.K. (2002). Offloading difficult wounds and conditions in diabetic patient. Ostomy/Wound Manag..

[27] Hemler S.L., Ntella S.L., Jeanmonod K., Köchli C., Tiwari B., Civet Y., Perriard Y., Pataky Z. (2023). Intelligent plantar pressure offloading for the prevention of diabetic foot ulcers and amputations. Front. Endocrinol..

[28] Erel V., Nasirian A., Gu Y., Lavery L., Wijesundara M.B.J. (2024). Development of Cyclic Pressure Offloading Insole for Diabetic Foot Ulcer Prevention. Int. J. Low. Extrem. Wounds.

[29] Castro-Martins P., Marques A., Pinto-Coelho L., Fonseca P., Vaz M. (2025). A Portable Insole System for Actively Controlled Offloading of Plantar Pressure for Diabetic Foot Care. Sensors.

[30] Formosa C., Borg A., Papanas N., Mizzi S. (2020). Adherence to Therapeutic Footwear in Type 2 Diabetes in Malta. Exp. Clin. Endocrinol. Diabetes.

[31] Ehrmann D., Spengler M., Jahn M., Niebuhr D., Haak T., Kulzer B., Hermanns N. (2018). Adherence Over Time: The Course of Adherence to Customized Diabetic Insoles as Objectively Assessed by a Temperature Sensor. J. Diabetes Sci. Technol..

[32] Couper M.P., Tourangeau R., Conrad F.G., Singer E. (2006). Evaluating the Effectiveness of Visual Analog Scales. Soc. Sci. Comput. Rev..

[33] Arceri A., Mazzotti A., Liosi S.G., Zielli S.O., Artioli E., Golinelli D., Brognara L., Faldini C. (2024). What’s the Impact of Safety Footwear on Workers Concerning Foot-Related Problems? A Systematic Review. Healthcare.

[34] Kelly A.M. (1998). Does the clinically significant difference in visual analog scale pain scores vary with gender, age, or cause of pain?. Acad. Emerg. Med..

